# MCP-4 and Eotaxin-3 Are Novel Biomarkers for Chronic Obstructive Pulmonary Disease

**DOI:** 10.1155/2023/8659293

**Published:** 2023-05-09

**Authors:** Chun Fang, Baoguo Kang, Pan Zhao, Jing Ran, Lifang Wang, Lingqiong Zhao, Hangyu Luo, Ling Tao

**Affiliations:** ^1^Department of Oncology, The First People's Hospital of Chongqing Liang Jiang New Area, Chongqing 401121, China; ^2^Department of General Surgery, The First People's Hospital of Chongqing Liang Jiang New Area, Chongqing 401121, China; ^3^Department of Pathology, The First People's Hospital of Chongqing Liang Jiang New Area, Chongqing 401121, China; ^4^Departments of Obstetrics and Gynecology, The First People's Hospital of Chongqing Liang Jiang New Area, Chongqing 401121, China; ^5^Department of Oncology, Chongqing General Hospital, Chongqing 400010, China; ^6^Department of Internal Medicine, The Chongqing Red Cross Hospital, Chongqing 400021, China

## Abstract

The aim of our study was to examine the production of monocyte chemoattractant protein (MCP-4) and eotaxin-3 during the onset and progression of COPD. The expression levels of MCP-4 and eotaxin-3 were evaluated in COPD samples and healthy controls using immunostaining and ELISA. The relationship between the clinic pathological features in the participants and the expression of MCP-4 and eotaxin-3 were evaluated. The association of MCP-4/eotaxin-3 production in COPD patients was also determined. The results revealed enhanced production of MCP-4 and eotaxin-3 in COPD patients especially the cases with AECOPD in both bronchial biopsies and bronchial washing fluid samples. Furthermore, the expression signatures of MCP-4/eotaxin-3 show high AUC values in distinguishing COPD patients and healthy volunteers and AECOPD and stable COPD cases, respectively. Additionally, the number of MCP-4/eotaxin-3 positive cases was notably increased in AECOPD patients compared to those with stable COPD. Moreover, the expression of MCP-4 and eotaxin-3 was positively correlated in COPD and AECOPD cases. In addition, the levels of MCP-4 and eotaxin-3 could be increased in HBEs stimulated with LPS, which is a risk factor of COPD. Moreover, MCP-4 and eotaxin-3 may exert their regulatory functions in COPD by regulating CCR2, 3, and 5. These data indicated that MCP-4 and eotaxin-3 were potential markers for the clinical course of COPD, which could provide guidance for accurate diagnosis and treatment for this disease in future clinical practice.

## 1. Introduction

Chronic obstructive pulmonary disease (COPD) is a common type of inflammatory lung disease, and the global prevalence was 251 million in 2016, which poses great threat to the public health system [1–3]. COPD is caused by the exposure to environmental pollution and primarily cigarette smoke, consequently leading to chronic airway inflammation [4], and the symptoms persist even after smoking cessation[5]. Previous reports revealed increased inflammatory markers in COPD such as C-reactive protein (CRP), interleukin-6 (IL-6), interleukin-8 (IL-8), tumor necrosis factor *α* (TNF-*α*), and YKL-40 [6–10]. Recent studies have suggested the essential roles of monocyte chemoattractant protein (MCP-4) and eotaxin-3 in the pathophysiology of airway inflammation [11–14]. MCP-4 is a member of a distinct, structurally related subclass of CC chemokines with activities on monocytes, eosinophils, and basophils. Eotaxin-3 belongs to a group of CC chemokines that attract eosinophils, basophils, and Th2 lymphocytes. Eotaxin-3 could promote local eosinophilic inflammation and is the most efficient eotaxin to induce the migration or transmigration of eosinophils [12, 13]. However, the expression profile and detailed regulatory function of these cytokines in COPD remain largely unknown.

The aim of this study was to evaluate the expression levels of MCP-4 and eotaxin-3 in patients with COPD and investigate whether the production of these cytokines was associated with the inflammation and development of this disease. Therefore, MCP-4 and eotaxin-3 could be novel markers of the disease progression, and targeted therapy against these cytokines may also be developed for the treatment of COPD patients in future clinical studies.

## 2. Methods

### 2.1. Clinical Specimens

A total of 47 COPD patients and 28 healthy volunteers were enrolled in this study between Jan 2012 and Oct 2015. Written informed consent forms were signed by all participants, and our protocol was approved by the Ethics Committee of the First People's Hospital of Chongqing Liang Jiang New Area, China. Airflow limitation was evaluated using spirometry and defined as post-bronchodilator FEV1/FVC < 0.70, and COPD was classified according to Global Initiative for Chronic Obstructive Lung Disease criteria [15]. Dyspnoea, cough, or sputum production was observed in all patients. Additionally, patients diagnosed with respiratory disorders other than COPD, such as asthma or bronchiectasis, were excluded in this study. All participants had stopped smoking for more than 2 months prior to this study. Patients with conditions known to be associated with increased cytokines in bronchial washing fluid, such as ALI/ARDS1 [16], were also excluded. The production of MCP-4 and eotaxin-3 was evaluated in all participants.

### 2.2. Criteria of Acute Exacerbation

Acute exacerbation was confirmed according to the existence of any two essential symptoms (dyspnoea, sputum purulence, or sputum quantity), or one major as well as one minor symptom (wheezing, sore throat, cough, or nasal congestion/discharge) for at least two days. Acute exacerbation was defined as deterioration in respiratory symptoms that requires antibiotics and/or systemic corticosteroid treatment, which are not required for the treatment of stable COPD.

### 2.3. Collection of Bronchial Biopsies and Bronchial Washing

Bronchoscopies were carried out in accordance with the relevant guidelines [17]. Bronchoscopy was performed on stable COPD patients following blood test and lung function test after 12 h fasting. Bronchoscopy was performed on AECOPD patients one day following admission before corticosteroids were given. Only moderate/mild exacerbation patients were included in the study. Patients did not receive any other medication before bronchoscopy. The upper airways were anaesthetized using lignocaine (Xylocaine®; Sweden), and then fiberoptic bronchoscopy (Olympus, Japan) and bronchial washing were performed nasally by wedging the bronchoscope into the segmental bronchus of the middle lobe. A total of 20 ml of 0.9% of sterile NaCl was injected and immediately aspirated. In order to remove any mucus and big cell debris, 5 ml of bronchial washing fluid was centrifuged at 400*g* for 10 min and 1500*g* for 30 min. Small cell debris was removed through ultracentrifugation at 12,000*g* for 2 min. Subsequently, the supernatant was collected and aliquoted. The samples were stored at −80°C until further use.

### 2.4. Immunohistochemistry Analysis

Immunostaining of MCP-4 and eotaxin-3 was performed on paraffin-embedded sections. The bronchial biopsies were dewaxed using xylene and rehydrated through a graded ethanol series and water. Antigen retrieval was carried out using microwave treatment in 10 mM sodium citrate for 15 min. Tissues were then incubated with FCS at room temperature for 30 min and with corresponding primary antibody against MCP-4 (1 : 100; cat. No. SC-271124, Santa Cruz Biotechnology) or eotaxin-3 (1 : 200; cat. No. MA5-23858, Thermo Fisher Scientific) in a humid chamber at 4°C overnight. The following day, the sections were washed using PBS and incubated with biotin labeled secondary antibody (1 : 50; Dako, UK). Subsequently, antigen was visualized by streptavidin-biotin-peroxidase system (ABC kit; Dako, UK). Quantification of staining intensity was performed using ImageJ software (version 1.46; NIH, Bethesda, MD, USA). The stained biopsies were visualized under microscope and digital images were captured with fixed settings. In brief, by comparing to the control group (secondary antibody alone), threshold of positive staining was defined using the eyedropper tool of ImageJ. Color signal above the threshold was considered as positive staining, and vice versa. For automated counting, the optical counts of positive staining were determined using the defined threshold. The staining results were presented as mean density (integrated optical density/area). Staining intensities in normal vs. COPD or stable COPD vs. AECOPD samples were analyzed using Student's *t*-test. The differences were considered statistically significant at *p* < 0.05.

### 2.5. Cell Culture and Treatments

Human bronchial epithelial cells (HBEs) were purchased from ATCC (Manassas, VA). Cells were cultured with RPMI-1640 medium (Life Technologies) supplemented with penicillin and streptomycin in a humid incubator at 37°C supplied with 5% CO_2_. When cells were ∼80% confluent, they were they were seeded onto a six-well plate at the density of 5 × 10^5^ cells/well. Cells were treated with LPS (Sigma-Aldrich, St. Louis, MO), and nontreated cells were used as control. For the dose-effect study, cells were treated with LPS at indicated concentrations (0, 0.01, 0.1, and 1.0 *µ*g/mL) and incubated for 24 h. For the time-effect study, LPS (0.1 *µ*g/mL) was added to the culture supernatant and cells were collected after 0, 2, 6, and 24 h. Bonferroni correction was used for multiple comparison. Then, culture supernatants were collected and stored at −20°C for ELISA, and cell pellets were stored at −80°C for mRNA extraction.

### 2.6. Measurement of Cytokine Production

The levels of MCP-4 and eotaxin-3 were determined in bronchial washing fluid samples/culture supernatants. In brief, the samples were centrifuged at 1500 rpm at 4°C for 5 min. Expression of MCP-4 and eotaxin-3 was measured using the abovementioned antibodies (1 : 50 for MCP-4 and 1 : 100 for eotaxin-3). Serial dilutions of recombinant standards (MCP-4: cat. No. 327-P4; eotaxin-3: cat. No. 653-E3, R&D Systems) and samples were conducted according to the protocol of manufacturers. The standard curves were generated using the recombinant standards. The levels of cytokines were quantified using the serial dilutions of recombinant falling within the linear range.

### 2.7. RNA Extraction and qRT-PCR

Cells were treated with serial concentrations of LPS for 24 h in the dose-effect experiments or treated with 0.1 *µ*g/ml LPS in the time-effect study, and pellets were collected 2, 6, and 24 h after treatment. Total RNA was extracted from cells using RNeasy mini kit (Qiagen). The concentration of RNA was determined by NanoDrop 1000 spectrophotometer (Thermo Fisher Scientific). Subsequently, SuperScript first-strand kit (Invitrogen) was used for reverse transcription, and then qPCR was performed using SYBR Green PCR Master Mix (Takara Biotechnology Co. Ltd.). Endogenous GAPDH was used as control. Forward and reverse primers used were as follows: MCP-4, 5′-GATCTCCTTGCAGAGGCTGAAG-3′ and 5′-TCTGGACCCACTTCTCCTTTGG-3′;eotaxin-3, 5′-GGGAGTGACATATCCAAGACCTG-3′ and 5′-CAGACTTTCTTGCCTCTTTTGGTA-3′; GAPDH, 5′-CATGGCCTTCCGTGTTCCTA-3′ and 5′-TACTTGGCAGGTTTCTCCAGG-3′. PCR reaction was performed on a GeneAmp 9700 system (Applied Biosystems) using the following program: 95°C for 10 min, followed by 40 cycles of 95°C for 15 s, 60°C for 20 s, and 72°C for 10 s. Amplified samples were subjected to agarose gel (1%) in TBE (0.01 M) and EDTA (0.001 M) containing ethidium bromide (2.0 lg/mL; all from Invitrogen). Then, bands were visualized using a UV transilluminator (ChemiDoc XRS System).

### 2.8. Protein Extraction from Bronchial Biopsies and Western Blotting

Endobronchial biopsies were snap-frozen and stored at −80°C. Samples were homogenized using ice-cold lysis buffer containing Tris-HCl (50 mmol/L), NaCl (150 mmol/L), EDTA (5 mmol/L), protease inhibitor cocktail (1x; Roche, Mannheim, Germany), phenylmethanesulfonyl fluoride (1 mmol/L), and Triton X-100 (1%). Then, samples were incubated on ice for 20 min and then centrifuged at 12,000 rpm at 4°C for 15 min. The supernatants were obtained, and protein concentration was measured by a Bio-Rad assay kit (Hertfordshire, UK). Protein samples were subjected to SDS-PAGE and then transferred to a PVDF membrane (EMD Millipore, Billerica, MA, USA). The membranes were blocked with TBST containing 5% skimmed milk for 1 h and subsequently incubated with primary antibody at 4°C overnight. The antibodies used were as follows: MCP-4 (1 : 1000; cat. No. SC-271124); eotaxin-3 (1 : 1000; cat. No. MA5-23858); CCR2 (1 : 2000, cat. No. ab273050, Abcam); CCR3 (1 : 1000; cat. No. 23HCLC, Thermo Fisher Scientific), CCR5 (1 : 2000; cat. No. ab7346, Abcam), and GAPDH (1 : 2000; cat. No. sc-365062, Santa Cruz). Next day, membranes were washed using TBST and were incubated with HRP-conjugated goat anti-mouse (1 : 5000; cat. No. ab6789, Abcam) or anti-rabbit (1 : 5000; cat. No. ab6721, Abcam) IgG for 1 h. Blots were detected using an ECL kit (Thermo Fisher Scientific), and protein signals were quantified using ImageJ.

### 2.9. Statistical Analysis

Data were presented as mean ± SEM based on at least three independent experiments. Difference in distribution of the two groups was analyzed using the *χ*^2^ test. Comparisons of difference were performed using Student's *t*-test or Mann–Whitney *U* test using GraphPad Prism Software (version 7; San Diego, CA).

Receiver operating characteristic (ROC) curve analysis was used to evaluate the power of MCP-4/eotaxin-3 expression signature in distinguishing COPD from healthy or AECOPD from stable COPD cases. To examine linear correlations between two variables, Pearson's correlation test was conducted when appropriate. *p* < 0.05 was considered to indicate a statistically significant difference.

## 3. Results

### 3.1. Expression Levels of MCP-4 and Eotaxin-3 Were Increased in COPD Patients

The effects of inflammatory cytokines on chronic inflammatory airway diseases such as COPD have been emphasized recently [18–20]. The numbers of T lymphocytes and production of proinflammatory cytokines are increased in airway during the development of COPD [5]. Although previous reports have elucidated the roles of MCP-4 and eotaxin-3 in COPD [21–23], the detailed mechanisms remained largely unknown. Therefore, we hypothesized that the production of inflammatory cytokines MCP-4 and eotaxin-3 is associated with the progression of COPD. In order to investigate this, bronchial biopsies and bronchial washing fluid samples from COPD patients and healthy donors were collected, and the expression levels of MCP-4 and eotaxin-3 were determined.

**Figure 1:**
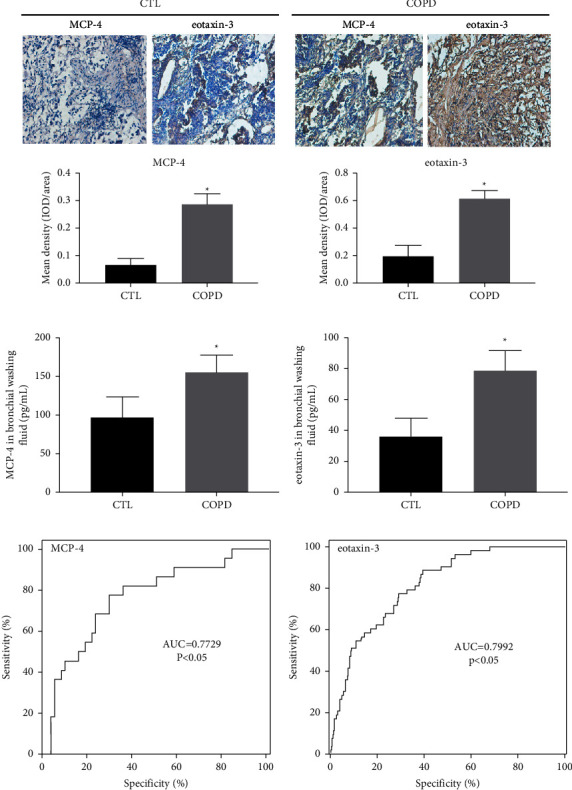
The expression levels of MCP-4 and eotaxin-3 were increased in COPD patients. (a, b) The presence of MCP-4 and eotaxin-3 in bronchial biopsies was revealed by immunostaining. The staining intensities of MCP-4 and eotaxin-3 were enhanced in the bronchial biopsies of patients with COPD compared to the healthy controls. (c, d) The production of MCP-4 and eotaxin-3 was elevated in the bronchial washing fluid samples obtained from COPD patients compared to the control group. (e, f) Receiver operating characteristic (ROC) curve revealed MCP-4/eotaxin-3 expression signature in distinguishing COPD patients and healthy controls. ^*∗*^*p* < 0.05 vs. healthy volunteers. COPD, chronic obstructive pulmonary disease; MCP-4, monocyte chemoattractant protein.

The characteristics of COPD patients and healthy volunteers are presented in Table 1. No significant difference in gender was observed between healthy volunteers and COPD patients. However, average age was remarkably increased in COPD group (*p* < 0.05). Body mass index (BMI) of all participants was within normal range, while that of COPD patients was relatively lower, but the difference was not statistically significant. Fourteen out of 47 COPD patients had no history of smoking, and their medical histories were reviewed. All fourteen patients were passive smokers. In addition, years of smoking and quitting time were remarkably increased in COPD patients (*p* < 0.05). In the results of immunostaining on bronchial biopsies, COPD patients exhibited significantly elevated production of MCP-4 and eotaxin-3 (Figures 1(a) and 1(b)). Furthermore, the levels of MCP-4 and eotaxin-3 were measured in bronchial washing fluid collected from COPD and control groups. Consistent with the findings of immunostaining, the results of ELISA suggested that production of MCP-4 and eotaxin-3 was remarkably enhanced in bronchial washing fluid samples from COPD patients (Figures 1(c) and 1(d)). ROC curve analyses indicated that MCP-4/eotaxin-3 expression signature exhibited high AUC value in distinguishing COPD patients and healthy controls, with sensitivity of 79.22/82.64% and specificity of 71.29/69.43%, respectively (Figures 1(e) and 1(f)).

### 3.2. Production of MCP-4 and Eotaxin-3 Was Upregulated in Patients with AECOPD Compared to Stable COPD

**Figure 2:**
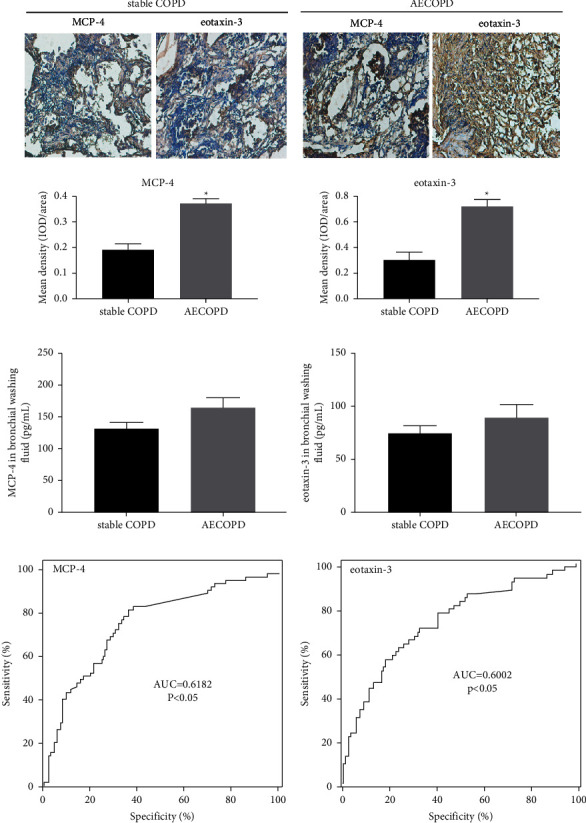
The production of MCP-4 and eotaxin-3 was enhanced in AECOPD patients. (a, b) The intensities of MCP-4 and eotaxin-3 staining were significantly increased in the bronchial biopsies of patients with AECOPD compared to those with stable COPD. (c, d) The expression levels of MCP-4 and eotaxin-3 were remarkably upregulated in bronchial washing fluid collected from AECOPD patients compared to stable COPD group. (e, f) ROC curve revealed MCP-4/eotaxin-3 production signature in distinguishing AECOPD patients and those with stable COPD. ^*∗*^*p* < 0.05 vs. stable COPD patients. COPD, chronic obstructive pulmonary disease; MCP-4, monocyte chemoattractant protein.

To study whether the production of MCP-4 and eotaxin-3 is correlated with the development of COPD, the production of these two cytokines was evaluated in bronchial biopsies and bronchial washing fluid samples from patients with AECOPD (*n* = 35) and those with stable COPD (*n* = 12). Thirty exacerbations were bacterial-associated and five were virus-related. A total of thirty patients were treated with antibiotics, and ten were treated with systemic corticosteroids. Twenty-two patients were supplied with oxygen during acute exacerbation. Duration of acute exacerbations ranged from 3 to 15 days. The clinical features of the participants are summarized in Table 2. There was no significant difference in gender between AECOPD and stable COPD groups. In addition, average age showed no difference in patients with AECOPD compared with stable COPD group. BMI of all participants was within normal range, and no significant difference was observed between the two experimental groups. Years of smoking exhibited no difference between AECOPD and stable COPD groups. Furthermore, there was no difference in respect to WBC, RBC, percentages of lymphocytes/neutrophils/eosinophils, and ESR, but CRP/IL-6 levels were remarkably increased in patients with AECOPD (*p* < 0.05). The data of immunostaining revealed elevated levels of MCP-4 and eotaxin-3 in AECOPD patients compared to those with stable COPD (Figures 2(a) and 2(b)). In addition, the results of ELISA were in agreement with these findings, as AECOPD patients exhibited increased production of MCP-4 and eotaxin-3 compared to stable COPD group (Figures 2(c) and 2(d)). Furthermore, MCP-4/eotaxin-3 expression signature also showed AUC values of 0.6182/0.6002 in differing AECOPD from stable COPD patients, with sensitivity of 38.26/39.33% and specificity of 76.42/78.66% (Figures 2(e) and 2(f)).

### 3.3. Association of MCP-4 and Eotaxin-3 Levels with Clinicopathological Features of COPD Patients

**Figure 3:**
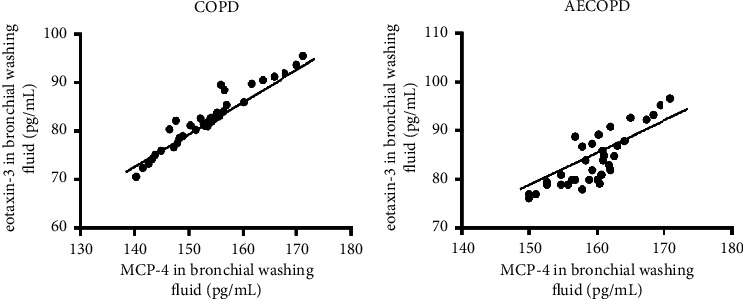
The association between the production of MCP-4 and production of eotaxin-3 in COPD patients and AECOPD cases. (a) The expression levels of MCP-4 and eotaxin-3 in the bronchial washing fluid of patients with COPD were positively correlated (*r* = 0.6857; ^*∗*^*p* < 0.05). (b) Positive correlation of MCP-4 and eotaxin-3 production was also detected in AECOPD patients (*r* = 0.6322; ^*∗*^*p* < 0.05). COPD, chronic obstructive pulmonary disease; MCP-4, monocyte chemoattractant protein.

The correlations between MCP-4 and eotaxin-3 production and the characteristics of patients with COPD were analyzed, and the results are presented in Table 3. No significant difference in age, gender, and smoking history was observed between MCP-4/eotaxin-3 positive and negative groups. In addition, the number of MCP-4/eotaxin-3 positive cases was significantly increased in AECOPD compared to stable COPD group (*p* < 0.05). Moreover, positive correlation between MCP-4 and eotaxin-3 production was revealed in the bronchial washing fluid samples of COPD patients, and the association was also observed in AECOPD cases (Figure 3; *r* = 0.679, *p* < 0.05; *r* = 0.625, *p* < 0.05), suggesting the correlation between MCP-4 and eotaxin-3 levels in the airway of COPD patients.

### 3.4. LPS Stimulated the Production of MCP-4 and Eotaxin-3 in HBEs

**Figure 4:**
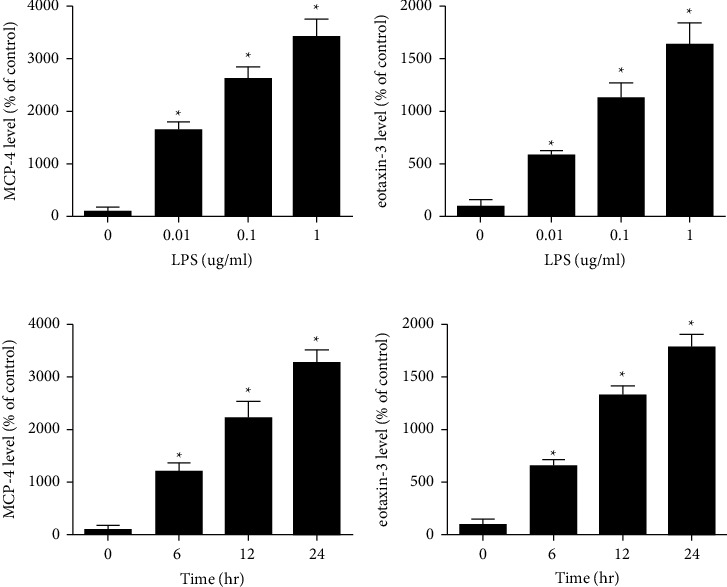
Protein levels of MCP-4 and eotaxin-3 were upregulated in HBEs stimulated with LPS. (a, b) Expression of MCP-4 and eotaxin-3 in culture medium of HBEs was increased after the treatment with LPS (0.01–1 *µ*g/mL) in a dose-dependent manner. (c, d) LPS-stimulated upregulation of MCP-4 and eotaxin-3 in HBEs exhibited a time-dependent manner. ^∗^*p* < 0.05. MCP-4, monocyte chemoattractant protein.

**Figure 5:**
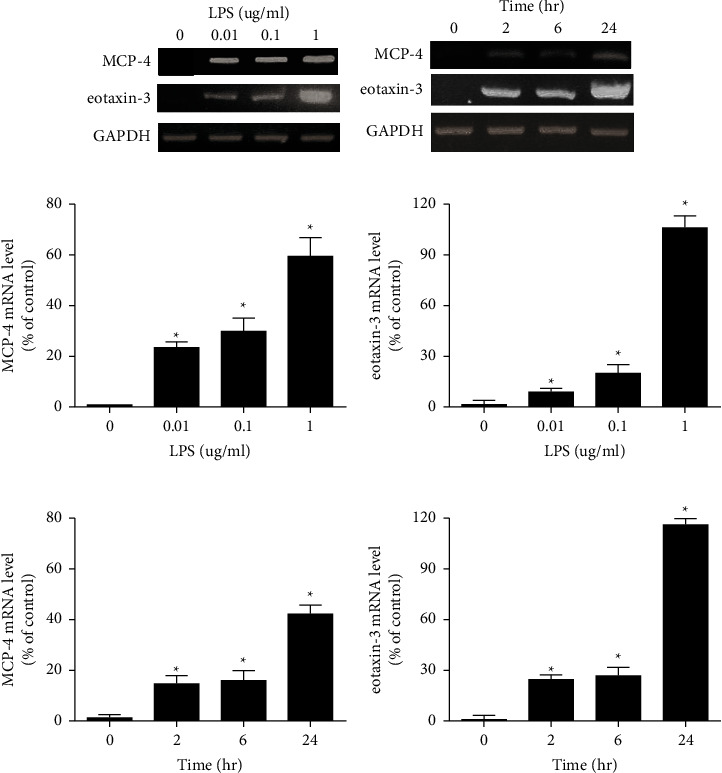
mRNA levels of MCP-4 and eotaxin-3 were elevated in HBEs treated with LPS. (a, b) Expression of MCP-4 and eotaxin-3 was elevated in HBEs stimulated with LPS. (c–f) LPS-induced increase of MCP-4 and eotaxin-3 mRNAs also exhibited dose- and time-dependent manner. ^∗^*p* < 0.05. MCP-4, monocyte chemoattractant protein.

In order to confirm that MCP-4 and eotaxin-3 are putative biomarkers for COPD, further function studies were performed on HBEs. The protein levels of MCP-4 and eotaxin-3 in the culture medium of HBEs were increased following the treatment with LPS (0.01−1 *µ*g/mL) for 24 h in a dose-dependent manner (Figures 4(a) and 4(b)). In addition, LPS-stimulated production of MCP-4 and eotaxin-3 in HBEs also exhibited a time-dependent manner (Figures 4(c) and 4(d)). Consistent with these findings, the mRNA levels of MCP-4 and eotaxin-3 were also elevated in HBEs stimulated with LPS (Figures 5(a) and 5(b)). In further dose- and time-effect studies, LPS-induced increase of MCP-4 and eotaxin-3 mRNAs also exhibited dose- and time-dependent manner (Figures 5(c)–5(f)).

### 3.5. MCP-4 and Eotaxin-3 May Exert Their Functions in COPD by Targeting Chemokine Receptor 2 (CCR2), 3 (CCR3), and 5 (CCR5)

**Figure 6:**
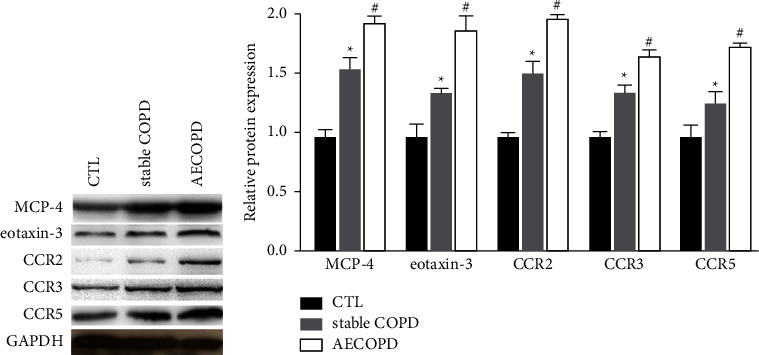
MCP-4 and eotaxin-3 could function through CCR2, 3, and 5 in COPD. Protein levels of MCP-4, eotaxin-3, and CCR2, 3, and 5 were examined in COPD and normal samples. The expression of abovementioned molecules was determined in AECOPD and stable COPD cases. COPD, chronic obstructive pulmonary disease; MCP-4, monocyte chemoattractant protein. ^*∗*^*p* < 0.05 vs. control; ^#^*p* < 0.05 vs. stable COPD.

Further experiments were conducted to investigate the novel downstream signaling of MCP-4 and eotaxin-3 in COPD. Previous studies suggested that MCP-4 serves essential roles in inflammation through its downstream molecules CCR2, 3, and 5 (24–26). In addition, eotaxin-3 could be a key regulator during the recruitment of eosinophils in asthma via activating CCR3 specifically (26). Therefore, the protein levels of MCP-4 and eotaxin-3 as well as their downstream molecules CCR2, 3, and 5 were evaluated in samples from COPD patients and healthy donors. The results indicated that the expression of MCP-4, eotaxin-3, and CCR2, 3, and 5 was significantly elevated in COPD group compared to normal control (Figures 6(a) and 6(b); *p* < 0.05). Furthermore, western blot analysis also revealed remarkable upregulation of MCP-4, eotaxin-3, and CCR2, 3, and 5 in AECOPD patients in comparison with those with stable COPD (Figures 6(a) and 6(b); *p* < 0.05).

## 4. Discussion

COPD is a type of inflammatory disorder in lung, and the inflammatory process is characterized with aberrant activation of both innate and adaptive immune responses in airway tracts [1–3]. The essential roles of cytokines including MCP-4 and eotaxin-3 in the pathogenesis of COPD have been previously revealed [4, 6–8]; however, the detailed function and underlying mechanisms are not completely understood. MCP-4 is involved in the recruitment of eosinophils, monocytes, and T-cells in mucosal inflammatory disorders such as asthma [23]. Enhanced production of MCP-4 has been detected in asthma samples especially in those with acute asthma exacerbations [23]. Eotaxin-3 is one of the eosinophil chemotactic factors released by bronchial and inflammatory cells, and it is involved in the recruitment and activation of eosinophils [13, 24, 25]. Recent study has revealed increased mRNA levels of eotaxin-3 in airway epithelial brushings of asthmatic patients [26]. This study was conducted to evaluate the expression levels of MCP-4 and eotaxin-3 in COPD patients and healthy controls. The association between the expression signatures of MCP-4/eotaxin-3 and clinicopathological features of patients was also investigated.

Our data indicated that the production of MCP-4 and eotaxin-3 was promoted in both bronchial biopsies and bronchial washing fluid samples obtained from COPD patients especially from those with AECOPD. The results suggested that the levels of MCP-4 and eotaxin-3 were significantly upregulated in the airway of AECOPD patients, indicating that the production of these two cytokines might predict the progression of COPD. In summary, upregulated MCP-4 and eotaxin-3 expression could be novel biomarkers for the development of COPD. Consistent with our findings, previous reports also revealed increased production of systemic MCP-4 in the serum samples from COPD patients [11, 14]. Similarly, the expression levels of eotaxin-3 were elevated in respiratory inflammatory diseases such as patients with asthma and AECOPD [12, 13, 27]. Furthermore, ROC curve analyses also suggested that MCP-4/eotaxin-3 expression signature exhibited high AUC value in distinguishing COPD patients and healthy controls. Additionally, the number of MCP-4/eotaxin-3 positive cases was remarkably elevated in AECOPD patients compared to those with stable COPD. Furthermore, the expression levels of MCP-4 and eotaxin-3 were positively correlated in both COPD and AECOPD groups. In addition, the production of MCP-4 and eotaxin-3 in HBEs could be stimulated by LPS, which is a key risk factor for COPD. Moreover, MCP-4 and eotaxin-3 may exert their regulatory functions in COPD through CCR2, 3, and 5. However, there were some limitations in this study. For example, the sample size was limited and large-scale study is required in future to confirm these findings. Additionally, previous study indicated that age might affect the production of cytokines/chemokines [28], which was not observed in this study. The correlation between age and MCP-4/eotaxin-3 production should be explored in future study using increased sample size and broadened age range.

In summary, our results suggested that the production of MCP-4 and eotaxin-3 could be associated with the development of COPD, and these cytokines could be putative indicator of the onset and progression of COPD. These findings may provide evidence to guide the diagnosis/treatment of this disease in future clinical practice.

## Figures and Tables

**Table 1:** Clinical features of COPD patients and healthy donors.

	Healthy controls (*n* = 28)	COPD patients (*n* = 47)	*p* value
Age	55.11 ± 2.46	73.11 ± 1.45	<0.05
Gender (male/female)	20/8	34/13	0.93
Body mass index (kg/m^2^)	22.61 ± 0.61	22.49 ± 0.37	0.29
Years of smoking	19.37 ± 3.52	26.82 ± 2.95	<0.05
Quitting time (year)	1.05 ± 0.68	6.65 ± 1.07	<0.05

**Table 2:** Characteristics of stable COPD and AECOPD patients.

	Stable COPD (*n* = 12)	AECOPD (*n* = 35)	*p* value
Age	73.01 ± 2.82	74.16 ± 1.66	0.09
Gender (male/female)	9/3	25/10	0.81
Body mass index (kg/m^2^)	21.13 ± 0.71	21.45 ± 0.42	0.07
Years of smoking	26.21 ± 4.33	27.66 ± 1.42	0.09
Quitting time (year)	6.28 ± 1.49	6.68 ± 0.82	0.25
WBC (×10^9^/L)	16.98 ± 3.01	18.95 ± 3.14	0.06
RBC (×10^12^/L)	4.52 ± 0.21	4.65 ± 0.30	0.17
Lymphocytes (%)	15.42 ± 3.46	14.45 ± 2.98	0.36
Neutrophils (%)	84.56 ± 8.32	87.87 ± 8.56	0.25
Eosinophils (%)	2.86 ± 0.29	3.09 ± 0.38	0.06
CRP (mg/L)	7.34 ± 3.29	15.58 ± 4.56	<0.05
IL-6 (pg/mL)	4.63 ± 2.02	6.05 ± 1.68	<0.05
ESR (mg/L)	27.01 ± 2.93	29.88 ± 6.22	0.13

**Table 3:** The association between MCP-4/eotaxin-3 production and clinical features of COPD patients.

	*n*	MCP-4 expression		*χ* ^2^	*p*	Eotaxin-3 expression		*χ* ^2^	*p*
		−	+			−	+		
Age				0.028	0.866			0.059	0.807
≥60	36	11	25			9	27		
<60	11	3	8			3	8		
Gender				0.053	0.818			0.003	0.956
Male	33	10	23			8	25		
Female	14	4	10			4	10		
Smoking history				0.200	0.655			0.018	0.892
Nonsmoker	13	5	8			3	10		
Smoker	34	9	25			9	25		
Disease severity				4.785	0.029			5.802	0.016
Stable	12	9	3			8	4		
AECOPD	35	5	30			4	31		

## Data Availability

The datasets used and/or analyzed during the current study are available from the corresponding author on reasonable request.
